# A unified framework to explore soliton boundary interaction using topological magnetic soliton spring oscillators

**DOI:** 10.1038/s41598-025-05241-4

**Published:** 2025-09-30

**Authors:** Shizhu Qiao, Yan Zhou, Shishen Yan, Zhiyong Quan, Wenjia Yang

**Affiliations:** 1 Department of Physics and Electronic Engineering, Jinzhong University, Jinzhong, 030619 China; 2 Guangdong Basic Research Center of Excellence for Aggregate Science, School of Science and Engineering, The Chinese University of Hong Kong, Shenzhen, 518172 China; 3 School of Physics, State Key Laboratory of Crystal Materials, Shandong University, Jinan, 250100 China; 4 Key Laboratory of Magnetic Molecules and Magnetic Information Materials of the Ministry of Education, Research Institute of Materials Science, Shanxi Normal University, Taiyuan, 030000 China

**Keywords:** Spintronics, Topological matter

## Abstract

Soliton-boundary interactions significantly influence the dynamics, stability, and functionality of topological magnetic solitons in spintronic devices, yet quantifying these interactions remains challenging. In this work, we introduce a unified framework termed the “topological magnetic soliton spring oscillator”, designed to systematically explore and quantify soliton-boundary interactions across different soliton types, including hopfions, skyrmions, and domain walls. Within this framework, soliton motion is governed by the competition between two effective forces: the spin-transfer torque-induced driving force​, and the boundary-induced repulsive force​, which mainly arises from excess exchange energy near boundaries. Through comprehensive micromagnetic simulations, we reveal that the interaction behavior transitions between linear and nonlinear regimes, depending on the degree of soliton deformation and the value of the damping factor. Specifically, for zero damping (*α* = 0), when the soliton deformation is minimal, the interaction energy exhibits a linear dependence on coordinates, while significant deformation induces nonlinearity with a slope increasing toward boundaries. For small damping factors, solitons exhibit damped oscillations with velocity-dependent, multivalued interaction energy. At larger damping, overdamped dynamics dominate, characterized by nonlinear interactions with a decreasing slope as the boundary is approached. This framework not only provides a physical basis for interpreting soliton-boundary behavior in topological magnetic systems but also identifies key dynamical features relevant to the design of soliton-based spintronic devices.

## Introduction

Topological magnetic solitons, such as hopfion^1–4^ skyrmion^5–9^ and domain wall^10–12^ have garnered significant attention in recent years due to their stable, particle-like behavior and potential applications in data storage^10,13–17^ artificial neuron network^18,19^ frequency comb^20,21^and other cutting-edge technologies^22–24^. A critical factor affecting the dynamics of topological magnetic solitons, and thus the performance of these devices, is the soliton-boundary interaction. This interaction plays a pivotal role in governing soliton motion within confined spaces, influencing key behaviors such as transformation, oscillation, and stability, all of which are crucial for efficient device performance^25–36^.

For instance, stripe domains may transform into skyrmions when they are compressed by the boundaries of a narrow channel^5^. Additionally, the detrimental transverse motion of skyrmions caused by the skyrmion Hall effect^37,38^ can be effectively mitigated by boundary-induced repulsion^29,39–41^. In magnetic multilayers, the skyrmion–boundary interaction has been shown to be tunable via interlayer exchange coupling^40^. In epitaxial Pd/Fe/Ir(111) systems, the addition of ferromagnetic Co/Fe rims at the boundary prevents skyrmion annihilation and enables the stabilization of skyrmions and target states even in zero field^41^. Moreover, theoretical and micromagnetic studies have established a critical boundary force that determines the threshold current density for skyrmion expulsion^29^.

Boundary repulsion also plays a pivotal role in sustaining stable oscillation dynamics in skyrmion oscillators^31,42–46^. In circular ferromagnetic nanodisks, introducing locally modified edge magnetization creates a tunable repulsive potential that confines the skyrmion within a bounded orbit^44^. In synthetic antiferromagnetic structures, the interplay between interlayer exchange coupling and boundary confinement enables coherent rotational dynamics of coupled skyrmions^43^. Additionally, in skyrmion–domain wall hybrid structures, repulsion between the skyrmion and domain wall serves as a spatially localized confining force. This interaction forms the basis of synchronized STNO arrays, where boundary repulsion not only stabilizes individual oscillators but also contributes to phase locking across multiple units, improving signal coherence and output power^47^​. Furthermore, in systems based on elongated skyrmions, boundary effects—together with engineered pinning sites—define an effective potential well that facilitates periodic deformation modes. The resulting oscillations are maintained by the balance between spin torque input, damping, and edge confinement, demonstrating the versatility of boundary-induced repulsion in supporting non-circular dynamic states^45^.

While the skyrmion-boundary interaction has been studied, the literature presents a somewhat confusing picture. Existing studies report a wide range of interaction energy types, including linear^34,48,49^ power-law^48^ harmonic^30,50^ and exponential^27,29,49,51^ with little consensus on the precise nature of this interaction. This variability raises important questions about the factors that determine the form and strength of the skyrmion-boundary interaction. In practical applications, controlling this interaction could be crucial for stabilizing skyrmion motion. However, the diversity in reported interaction energies suggests that different skyrmion states or device geometries may respond differently.

**Fig. 1:**
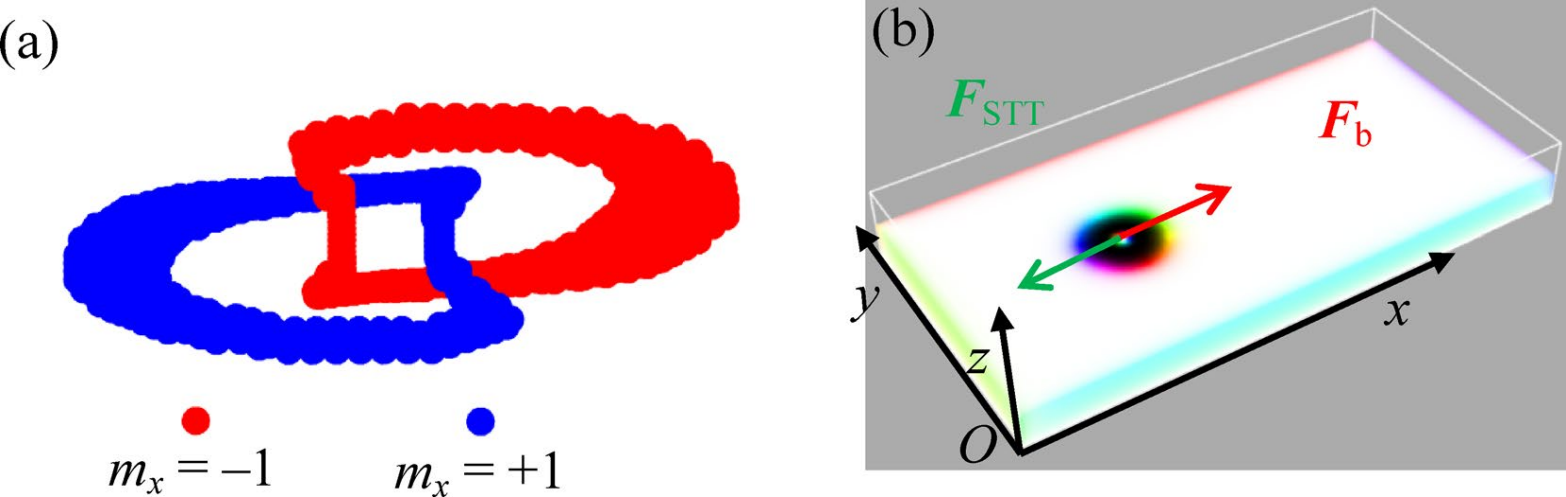
(**a**) The interlinking of contours corresponding to *m*_*x*_ = + 1 (blue) and *m*_*x*_ = − 1 (red), demonstrating the topological nature of the hopfion with a linking number of unity. (**b**) Magnetic hopfion profile at the mid-layer in the computational domain, with the coordinate axes used in this study indicated as *x*, *y*, and *z*. ***F***_STT_ and ***F***_b_ represent the effective forces acting on the hopfion, originating from spin-transfer torque and boundary repulsion, respectively.

Moreover, while skyrmion-boundary interactions have been somewhat explored, no explicit formulation for the quantized interaction energy between magnetic hopfions and boundaries has been proposed. Hopfions, being three-dimensional (3D) topological solitons with linked vortex rings, as depicted in Fig. 1(a), exhibit unique topological features that differentiate their dynamics from those of skyrmion^52,53^. Understanding the hopfion-boundary interaction is particularly crucial, as it could offer new insights into 3D soliton confinement and stability in nanoscale devices^1,26^.

Given the lack of a clear framework for quantifying this soliton-boundary repulsion, there is a promising avenue for developing a unified theory or model to describe these interactions in different contexts. To address this gap, we propose the concept of the topological magnetic soliton spring oscillator, drawing an analogy between the dynamics of solitons under boundary repulsion and the classical spring oscillator model. By introducing this framework, we aim to clarify the role of soliton-boundary repulsion, specifically focusing on the behaviors of hopfion, skyrmion, and domain wall in confined geometries.

Our results reveal that three key parameters govern the soliton dynamics and the nature of soliton-boundary interactions in these systems. The first is the Gilbert damping factor *α*, which characterizes energy dissipation and strongly influences the soliton’s oscillation amplitude and relaxation time. The second is the spin-transfer torque (STT) coefficient, which provides the driving force for soliton motion under an applied current. The interplay between STT and boundary-induced repulsion determines the oscillatory characteristics of the soliton, as depicted in Fig. 1(b). Finally, the geometric confinement of the system, including the sample size and boundary conditions, significantly impacts the strength and form of soliton-boundary interactions^25^. These geometric factors can modulate the energy landscape experienced by the soliton and thus shape its dynamic response to external stimuli.

This model not only offers a new framework on soliton-boundary interactions but also extends the classical oscillator concept to the realm of topological magnetic solitons, offering a versatile tool for exploring soliton dynamics and informing the design of advanced spintronic devices.

## Results of Hopfion

Our study on magnetic hopfion-boundary interaction is conducted by mumax3^54^ within a cuboid simulation box of dimensions 128 × 64 × 16 nm^3^ containing a single Bloch hopfion, as illustrated in Fig. 1(b). The coordinate system is established such that the *x*-coordinate of the hopfion center is defined as the distance between the hopfion center and the boundary at *x* = 0. When subjected to a spin-polarized current along the *x*-direction, the hopfion moves accordingly. Although a Hall effect for magnetic hopfion may occur^4,37^ the constrained dimensions of the cuboid along the *y*-axis suppress this possibility.

**Fig. 2:**
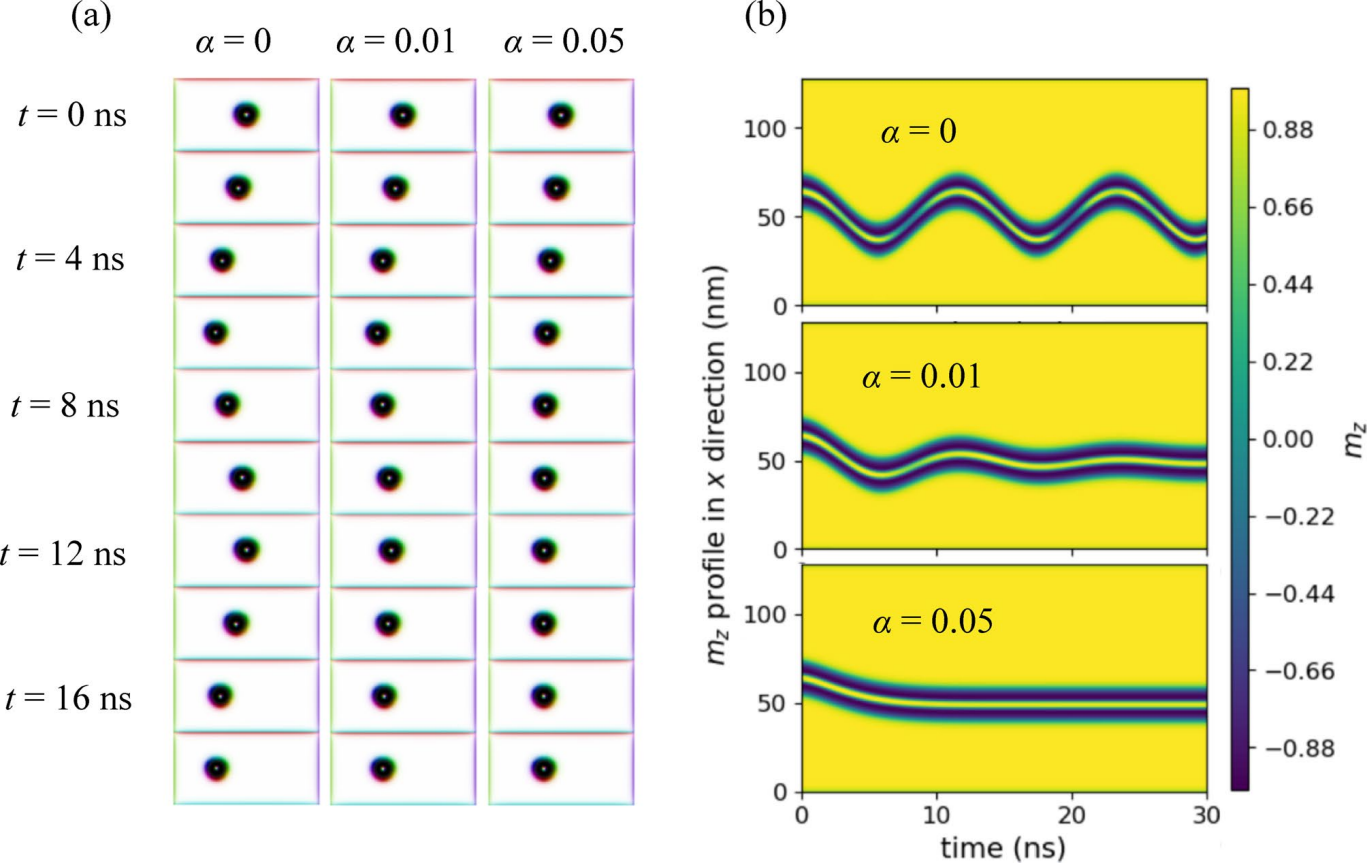
(**a**) Oscillatory motion of a magnetic hopfion subjected to spin-transfer torque and hopfion-boundary interaction. Three cases are shown, corresponding to Gilbert damping factors of *α* = 0, 0.01, and 0.05 represented in the left, middle, and right columns, respectively. (**b**) Time evolution of the *m*_*z*_ profile along the *x*-direction at *y* = 32 nm and *z* = 8 nm, for the same damping factors (*α* = 0, 0.01, and 0.05). A single time axis and color legend are shared across all three panels for clarity and conciseness, as the time range is identical for all cases.

Figure 2 illustrates the behavior of a magnetic hopfion interacting with boundaries under the influence of STT for three different Gilbert damping factors: *α* = 0, *α* = 0.01, and *α* = 0.05, shown in the left, middle, and right columns of Fig. 2(a), respectively. A constant current density of 4 × 10^9^ A/m^2^ is applied, and the hopfion’s movement is tracked at time intervals of 0 ns, 2ns, 4 ns, …, 16 ns, and 18 ns. The evolution of *m*_*z*_ along *x*-direction at *y* = 32 nm and *z* = 8 nm is depicted more clearly in Fig. 2(b). For *α* = 0, the hopfion is observed to move towards the boundary at *x* = 0 due to STT and then bounced back, continuing this back-and-forth motion akin to a spring oscillator. When *α* = 0.01, the hopfion exhibits a similar bouncing behavior, but with a gradually decreasing amplitude, indicative of energy dissipation over time due to nonzero damping, analogous to a damped spring oscillator. In the case of *α* = 0.05, no significant bouncing is observed, suggesting that the higher damping fully suppresses the hopfion’s ability to rebound, resembling the behavior of an overdamped spring oscillator.

To analyze hopfion-boundary interaction, we begin with the Landau-Lifshitz-Gilbert (LLG) equation, incorporating the STT terms in the Zhang-Li form^2,4,29^,

1 $$\frac{{{\text{d}}\varvec{m}}}{{{\text{d}}t}}=\gamma \left( {\varvec{m} \times {\varvec{B}_{{\text{eff}}}}} \right)+\alpha \left( {\varvec{m} \times \frac{{{\text{d}}\varvec{m}}}{{{\text{d}}t}}} \right)+\frac{{p{a^3}}}{{2e}}\left[ {(\varvec{j} \cdot \nabla )\varvec{m}} \right] - \frac{{p{a^3}\beta }}{{2e}}\left[ {\varvec{m} \times (\varvec{j} \cdot \nabla )\varvec{m}} \right]$$.

Here, ***m*** represents the unit vector characterizing the local magnetization, while the effective magnetic field ***B***_eff_​ is given by ${\varvec{B}_{{\text{eff}}}}=\left( { - 1/{M_{\text{s}}}} \right) \cdot {{\delta U} \mathord{\left/ {\vphantom {{\delta U} {\delta \varvec{m}}}} \right. \kern-0pt} {\delta \varvec{m}}}$, where *M*_s_ is the saturation magnetization and *δU*/*δ****m*** denotes the variational derivative of the free energy *U* with respect to ***m***. The parameters *γ*, *α*, *p*, *a*, *e*, ***j***, and *β* represent the gyromagnetic ratio, Gilbert damping factor, spin polarization of current, lattice constant, charge of electron, current density, and non-adiabatic STT coefficient, respectively.

Using Thiele’s approach^55,56^ velocity of hopfion along *x* direction reads as,

2 $${v_x}=\frac{{p{a^3}\beta j}}{{2e\alpha }} - \frac{\gamma }{{\alpha {M_{\text{s}}}{\Gamma _{xx}}}}\frac{{\partial U}}{{\partial x}}$$.

Details of derivation are listed in Supplementary Information. In this equation, the first term represents the contribution of the STT, which drives the hopfion’s motion along the *x*-direction^3,4^. The second term is associated with the gradient of the free energy along the *x*-axis, inducing a motion driven by variations in the energy landscape. ${\Gamma _{xx}}=\int {{\partial _x}{\varvec{m}} \cdot } {\partial _x}{\varvec{m}}{\text{d}}V$, which could be used to quantify the deformation of the hopfion induced by the STT and the repulsive interaction between hopfion and boundaries.

From the perspective of the magnetic hopfion, the total free energy *U*(*x*) can be decomposed into two components: *U*_H−B_(*x*) and *U*_0_(*x*). Here, *U*_H−B_(*x*) characterizes the repulsive interaction between the hopfion and the two boundaries perpendicular to the *x*-axis^25^ while *U*_0_(*x*) accounts for the energy of the hopfion that is independent of its distance to the boundaries. This decomposition helps to isolate and analyze *U*_H−B_(*x*) from the total energy.

To calculate *U*_H−B_(*x*), we choose the equilibrium position *x*_0_ (with *x*_0_ ​= 64 nm in this work) as the energy reference point, where the hopfion is sufficiently far from the boundary and thus the boundary repulsion vanishes, i.e., *U*_H−B_(*x*_0_) = 0. Consequently, at *x*_0_​, the total energy reduces to *U*_0_​(*x*_0_​). For convenience of calculation, we set *U*_0_​(*x*_0_​) = 0, so that the repulsive energy *U*_H−B_(*x*) can be directly extracted from the total energy *U*(*x*) using the following relation:

3 $${U_{{\text{H-B}}}}\left( x \right)=U\left( x \right) - {U_0}\left( x \right)=\int\limits_{{{x_0}}}^{x} {\left( {\frac{{p{a^3}\beta j}}{{2e}} - \alpha {v_x}} \right)\frac{{{\Gamma _{xx}}{M_{\text{s}}}}}{\gamma }{\text{d}}x} - {U_0}\left( x \right)$$,

*U*_0_​(*x*_0_​) is set to zero without loss of generality, because it only shifts the energy baseline but does not influence the force or dynamics governed by ∂*U*/∂*x*. The calculation of *U*_0_​(*x*​) is provided in detail in the Supplementary Information.

**Fig. 3:**
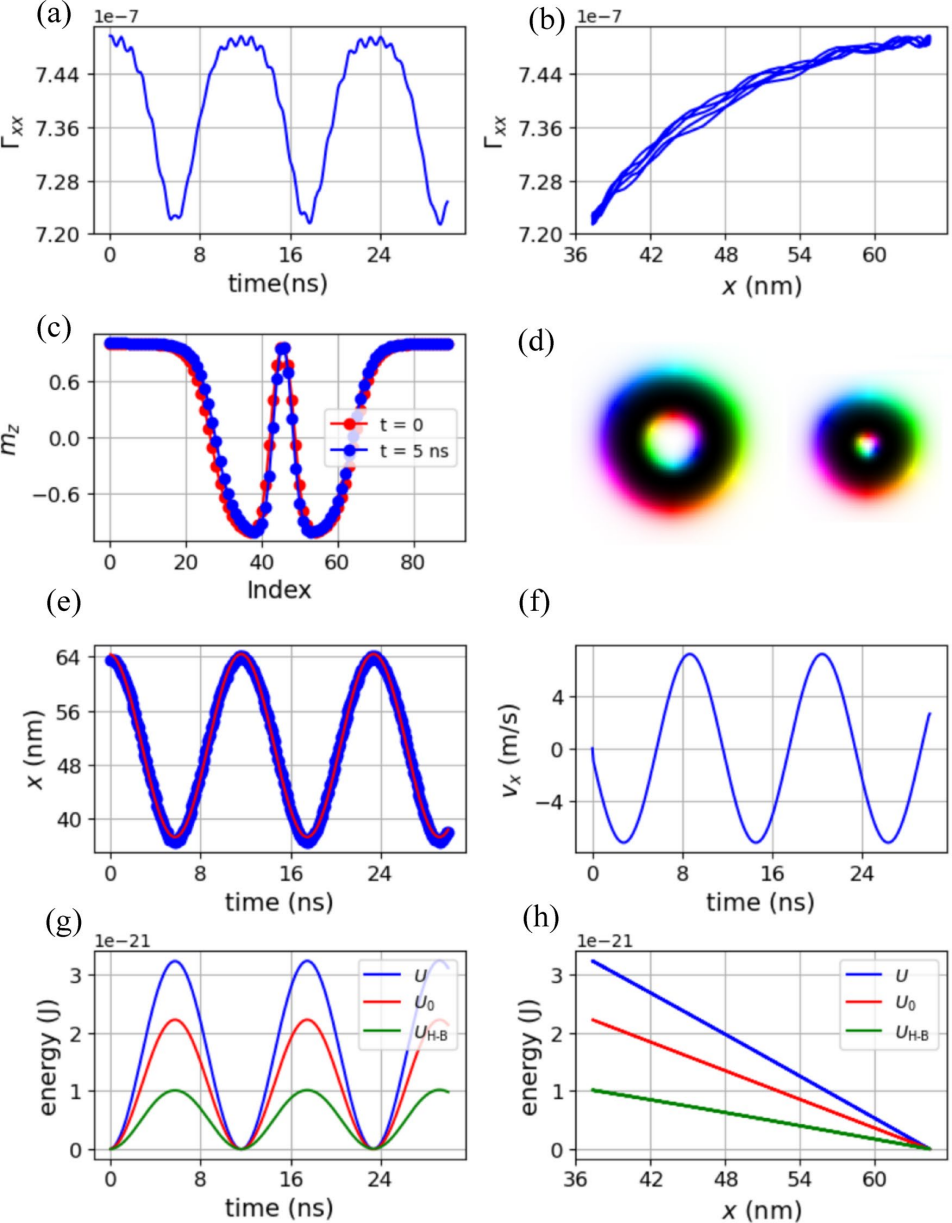
Numerical results for the case with *α* = 0: (**a**) Evolution of Γ_*xx*_ over time, (**b**) Γ_*xx*_ as a function of the *x*-coordinate of the hopfion center, showing how it varies with the hopfion’s position. (**c**) *m*_z_ profile of the magnetic hopfion along *x* direction at *y* = 32 nm, *z* = 8 nm for *t* = 0 ns and *t* = 5 ns, showing the temporal change in the magnetic texture. (**d**) Relative size comparison of the hopfion at the middle layer (*z* = 8 nm) of *L*_*y*_ = 128 nm on the left and *L*_*y*_ = 64 nm on the right. (**e**) Evolution of the *x*-coordinate of the hopfion center at layer *z* = 8 nm, fitted to a cosine function indicating a simple harmonic motion. (**f**) Velocity of the hopfion center corresponding to the fitted cosine function. (**g**) Evolution of the free energy *U*, *U*_0_, and *U*_H−B_ = *U* − *U*_0_ over time. *U*_H−B_ denotes the repulsive interaction energy between the hopfion and the boundaries perpendicular to the *x*-axis, while *U*_0_ represents the energy of the hopfion, which is independent of its distance from the boundary. (**h**) Dependence of *U*, *U*_0_, and *U*_H−B_ on the *x*-coordinate of the hopfion center.

The case with a Gilbert damping factor *α* = 0 is characterized in Fig. 3. The dependence of Γ_*xx*_ on time and on the *x*-coordinates of hopfion center are depicted in Fig. 3(a and b), where a variation of approximately 4% over one period can be observed. The serrations in Fig. 3(a) are caused by the breathing mode of the hopfion^52^. See Supplementary Information for more information. This, along with the limited simulation resolution, explains the irregularities observed in Fig. 3(b). The mismatch arises because the period of the hopfion’s motion along the *x*-axis is not an integer multiple of its breathing motion period. As mentioned earlier, Γ_*xx*_ serves as a metric for characterizing the deformation of the magnetic hopfion, induced by boundary repulsion and STT, which is further illustrated in Fig. 3(c). Here, two *m*_*z*_ profiles near the hopfion center, along the *x*-direction at *y* = 32 nm and *z* = 8 nm, are displayed for *t* = 0 ns and *t* = 5 ns. The horizontal axis labeled “index” refers to the grid index along the *x*-direction near the center of the hopfion. To facilitate comparison between *t* = 0 and *t* = 5 ns, we applied a shift to align the hopfion center, and therefore only relative index values are shown in the plot. These profiles correspond to two scenarios: the longest and shortest distances between the magnetic hopfion and the boundary at *x* = 0. A noticeable deformation of about 10% in the hopfion radius is observed between the two cases.

The effect of boundary repulsion is further highlighted in Fig. 3(d), which compares the relative sizes of two magnetic hopfions at the middle layer (*z* = 8 nm) within cuboids of different sizes: 128 × 128 × 16 nm^3^ on the left and 128 × 64 × 16 nm^3^ on the right. The smaller hopfion on the right indicates more intense repulsion, as expected, due to the reduced *L*_*y*_ dimension.

The motion of the hopfion is depicted in Fig. 3(e). The blue points represent the *x*-coordinates of the hopfion center, taken from the middle of the yellow line shown in Fig. 2(b), while the red line represents a cosine function fitted to the data. This fitting demonstrates that the motion of the magnetic hopfion closely resembles harmonic oscillation. From this fitting function, the velocity of the hopfion center, *v*_*x*_, can be determined, as shown in Fig. 3(f).

With both Γ_*xx*_ and *v*_*x*_ ​ in hand, one can calculate the hopfion-boundary interaction energy *U*_H−B_ ​using Eq. (3). The dependence of *U*, *U*_0_, and *U*_H−B_ on time and the *x*-coordinate of the hopfion center is illustrated in Fig. 3(g) and (h). The time dependence of *U*_H−B_, along with *U* and *U*_0_, exhibits harmonic behavior, much like the elastic potential energy *U*_EP_ ​ of a spring oscillator. However, one key difference is that the oscillation period of *U*_H−B_ ​ matches the period of the *x*-coordinate of the hopfion center, whereas the oscillation period of *U*_EP_ ​ in a standard spring oscillator is twice that of the *x*-coordinate.

Another significant difference is observed in Fig. 3(h), where the hopfion-boundary interaction energy appears linear in this magnetic hopfion spring oscillator, in contrast to the parabolic potential typical of an ordinary spring oscillator. One might argue that the linear appearance of the energy is due to the limited range of motion along the *x*-direction. However, numerical simulations on a cuboid of size 256 × 64 × 16 nm^3^ still exhibit a linear behavior. Refer to Supplementary Information for more details.

**Fig. 4:**
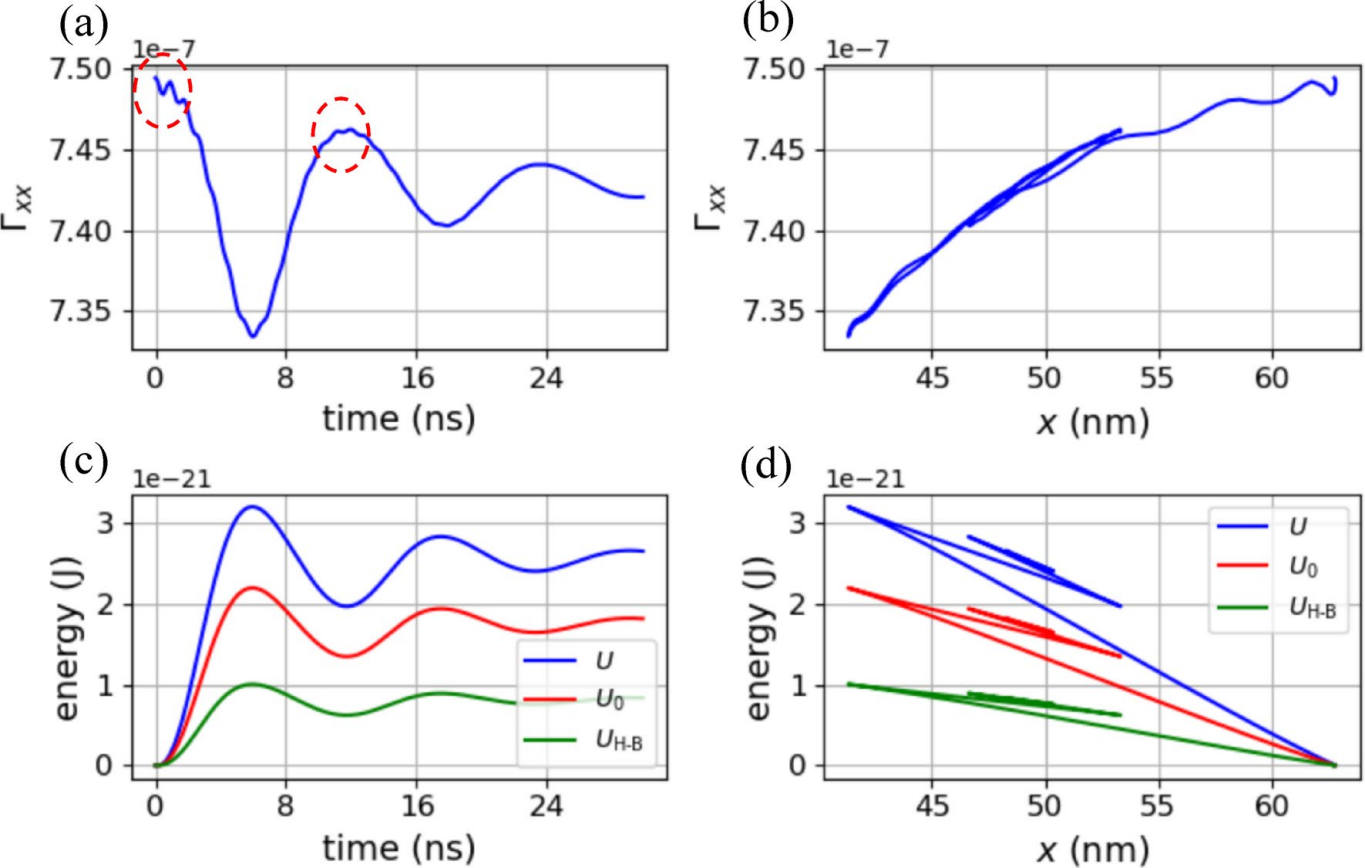
Numerical results of circumstance with *α* = 0.01: Dependence of Γ_*xx*_ on (**a**) time, and (**b**) *x* coordinate of hopfion center. (**c**) Dependence of *U*, *U*_0_, and *U*_H−B_ = *U* − *U*_0_ on time. *U*_H−B_ denotes the repulsive interaction energy between the hopfion and the boundaries perpendicular to the *x*-axis, while *U*_0_ represents the energy of the hopfion, which is independent of its distance from the boundary. (**d**) Dependence of *U*, *U*_0_, and *U*_H−B_ on *x* coordinate of hopfion center.

**Fig. 5:**
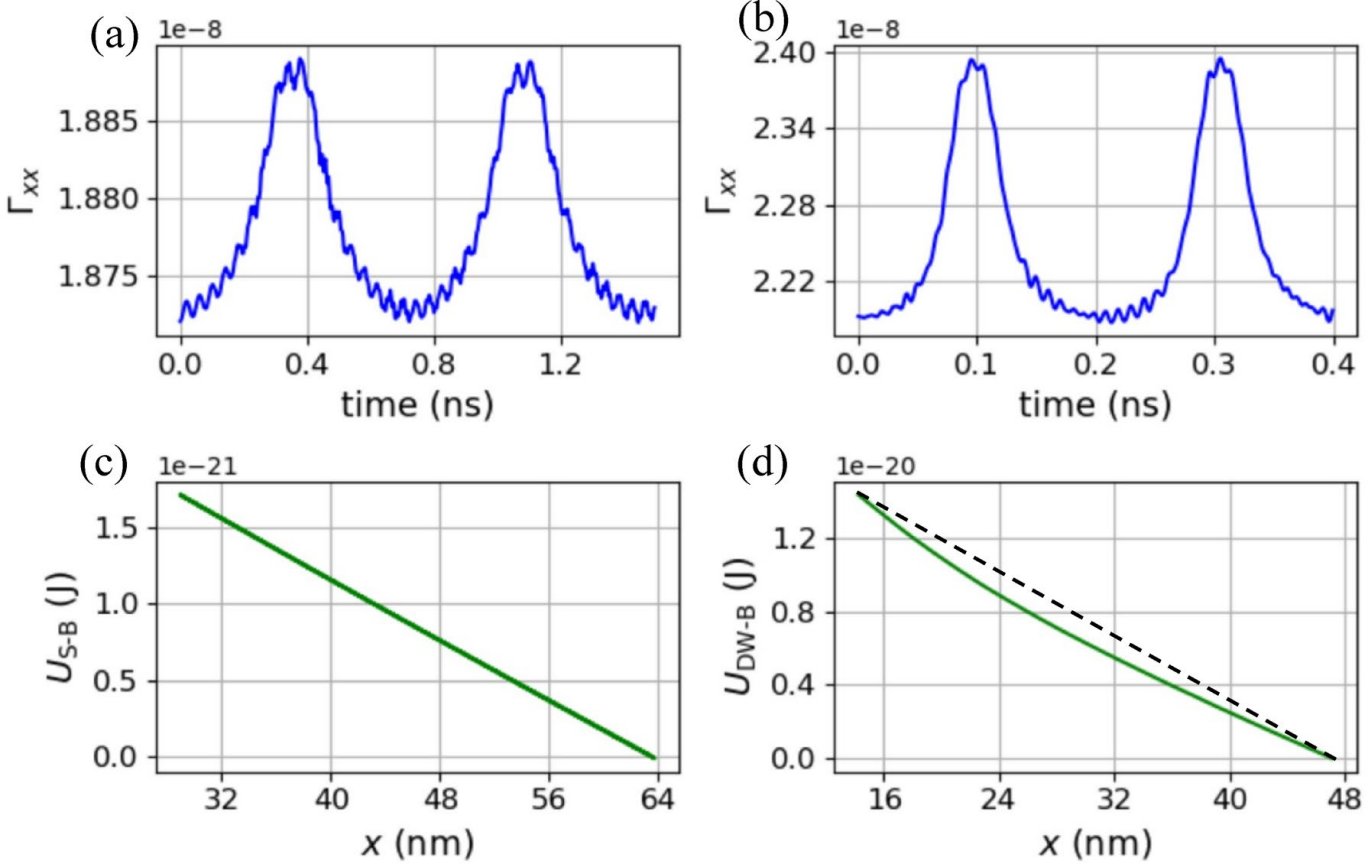
Numerical results of Néel skyrmion and domain wall in a synthetic antiferromagnet are shown in the left and the right columns, respectively. (**a**) and (**b**) depict the time dependence of Γ_*xx*_. (**c**) and (**d**) show the dependence of skyrmion-boundary energy, *U*_S−B_, and domain wall-boundary energy, *U*_DW−B_ on the *x*-coordinate of the soliton, respectively. A linear dash line is plotted in (**d**) for visual assistance.

This linearity could be explained in the following way. For the case of *α* = 0, one can derive the following equation from Eqs. (2),

4 $$\frac{{{{\partial U} \mathord{\left/ {\vphantom {{\partial U} {\partial x}}} \right. \kern-0pt} {\partial x}}}}{{{\Gamma _{xx}}}}=\frac{{p{a^3}\beta {M_{\text{s}}}}}{{2\gamma e}}j$$,

which suggests that if Γ_*xx*_ varies by less than 4%, ${{\partial U} \mathord{\left/ {\vphantom {{\partial U} {\partial x}}} \right. \kern-0pt} {\partial x}}$ should also exhibit a variation of less than 4%, for a given current density *j*. This could explain the linear energy observed in Fig. 3(h). However, if Γ_*xx*_ varies significantly, nonlinearity may be expected, as shown in Fig. 5(d). Additionally, if *α* ≠ 0, linear dependence may also disappear, as demonstrated in Fig. 4(d) for *α* = 0.01, and Supplementary Fig. S3 for *α* = 0.05 in Supplementary Information.

Another issue in Fig. 3(h) is that the smallest distance between the hopfion and the boundary appears insufficiently small, with *x*-coordinates larger than 36 nm in this scenario. This could potentially be addressed by increasing the current density. However, high current density might lead to the destruction of the hopfion. If the current density exceeds the value used in the simulation (e.g., 5 × 10^9^ A/m^2^), annihilation of the hopfion center may occur, leading to a topological transition from a hopfion to a toron^25,53^. For more details, refer to Supplementary Movie 3.

The scenario with a Gilbert damping factor *α* = 0.01 is illustrated in Fig. 4. In Fig. 4(a), Γ_*xx*_ demonstrates that the deformation of the hopfion oscillates with a decreasing amplitude and eventually stabilizes at a constant value. The serrations in two red dash circles indicate breathing mode, which is decreasing due to damping, leading to the decrease in irregularities shown in Fig. 4(b), in comparison to Fig. 3(b). The time evolution of *U*, *U*_0_, and *U*_H−B_, as depicted in Fig. 4(c), exhibits a pattern of decreasing amplitude, similar to the behavior of Γ_*xx*_.

Figure 4(d) reveals a noticeable multi-valued functions of *U*_H−B_ with respects to *x* coordinate of hopfion center, indicating that, the repulsive energy between the hopfion and the boundary is not solely dependent on their distance but also influenced by other parameters, such as the velocity of hopfion. This is supported by the numerical results from the integration in Eq. (3), where ruling out the contribution from *v*_*x*_ ​eliminates the multi-valued behavior. The velocity dependence is why we use the term “interaction energy” rather than “potential energy” to characterize soliton-boundary interaction. Furthermore, the velocity-dependent, multi-valued energy suggests that the hopfion oscillator exhibits memory effects, which could be useful for reservoir computing applications^18,19^.

### Results of skyrmion and domain wall

Equation (1) to (4) do not rely on a specific form of the magnetic texture, making this framework potentially applicable to other topological magnetic solitons, such as skyrmions, domain walls, and vortex. Numerical results for Néel skyrmion and domain wall in a synthetic antiferromagnet with *α* = 0 are depicted in the left and right columns of Fig. 5, respectively. Details of the simulation are listed in Supplementary Information. As shown in Fig. 5(a and b), the absolute values of Γ_*xx*_ for these two oscillators are significantly smaller than that of the hopfion in Fig. 3(a), due to their 2D nature. However, Γ_*xx*_ ​ of the domain wall oscillator in Fig. 5(b) varies significantly, by about 10%, suggesting a nonlinear soliton-boundary interaction, as indicated in Fig. 5(d). In contrast, the Γ_*xx*_ ​ of the skyrmion oscillator fluctuates by less than 1%, making the linear dependence observed in Fig. 5(c) unsurprising. Additionally, breathing modes are clearly indicated by the serrations in Fig. 5(a and b)^57,58^.

## Discussion

**Fig. 6:**
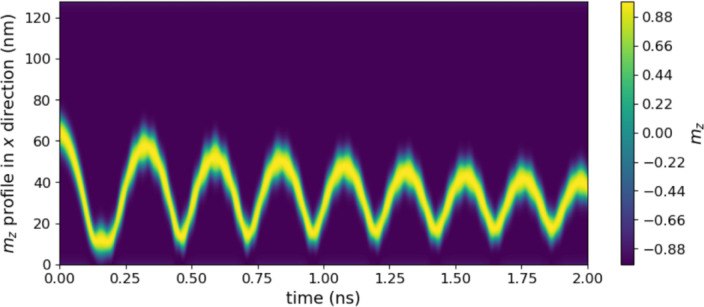
Time evolution of *m*_*z*_ profile in *x* direction at *y* = 32 nm of one sublayer of skyrmion spring oscillator with *α* = 0.001 driven by *j* = 10.00031 × 10^11^ A/m^2^.

Our oscillator models differ from previously reported topological magnetic soliton oscillators in at least two keyways. First, earlier oscillators, primarily based on skyrmion^31,42,43,46,59,60^ are inherently 2D. In contrast, our models are 1D, highlighting the effects of boundary repulsion and soliton deformation. Second, stable oscillation can occur in the previously reported models when  ≠ 0. However, in our models, stable oscillation appears to occur only when *α* = 0. For example, in simulations of the skyrmion oscillator with *α* = 0.001, we found that when the current density reached *j* = 10.00032 × 10^11^ A/m^2^, the skyrmion annihilates at boundary *x* = 0, as indicated by Supplementary Movie 4. In contrast, when *j* = 10.00031 × 10^11^ A/m^2^, a noticeable decrease in oscillation amplitude was observed, as depicted in Fig. 6.

A simplified explanation of this phenomenon can be provided using the concept of quasiparticles. In the 1D model, the STT does positive work when driving the soliton forward. Conversely, when the soliton bounces back, the STT performs negative work. This process is analogous to a vertically hanging spring oscillator, where gravity alternately performs positive and negative work. Consequently, any nonzero damping factor inevitably leads to a gradual reduction of oscillation amplitude. However, in the 2D model, this alternating work mechanism does not occur. Instead, STT continuously performs positive work, enabling sustained motion of the soliton even when damping is present^43,44^.

These findings highlight the significant differences between 1D and 2D oscillators. Consequently, we propose the term “topological magnetic soliton spring oscillator (TMSSO)” for our models. In TMSSO, STT acts analogously to gravity in a vertically hanging spring oscillator, and the magnet containing the topological soliton functions as the spring.

Beyond the current study, the proposed TMSSO framework offers promising opportunities for further exploration and applications. In particular, extending this framework to other topological solitons, such as torons^25^ or magnetic vortex rings^26^ could reveal soliton-boundary interaction arising from their distinct internal structures and dimensionalities. Moreover, recent studies have highlighted that confined geometries play a critical role in determining the stability, deformation, and transformation of topological magnetic solitons, especially for three-dimensional structures like hopfions and vortex rings^25,26^. For instance, Gao et al. demonstrated that the boundary-induced repulsion can drive the transformation of hopfions into torons in nanodisks with specific geometries. In addition, their work revealed topological transitions between hopfions and torons in finite-sized nanostripes and stepped nanostructures. Inspired by these results, future research could explore the application of the TMSSO framework in more complex or engineered geometries to achieve controllable soliton dynamics and enhanced stability. Such studies would further advance the understanding of soliton-boundary interactions and provide new strategies for the design of geometry-engineered devices.

## Conclusion

In conclusion, we introduce the concept of the topological magnetic soliton spring oscillator, establishing a unified framework for exploring soliton-boundary repulsion in confined geometries. This framework provides critical insights into the mechanisms governing soliton motion, highlighting its dependence on the free energy gradient and soliton deformation, and is applicable to various topological solitons. By analyzing soliton dynamics within this model, we demonstrate that when the Gilbert damping factor *α* = 0, linear soliton-boundary energy arises when soliton deformation due to boundary repulsion and spin-transfer torque is minimal. Significant deformation, however, leads to nonlinear interactions with an increasing slope as the soliton approaches the boundary. For small *α*, damped soliton oscillations result in multivalued soliton-boundary energy, revealing its velocity dependence and memory effects. In contrast, for large *α*, overdamped oscillations produce nonlinear interactions with a decreasing slope near the boundary.

This concept provides a versatile theoretical framework for quantifying soliton-boundary interactions and examining soliton deformation, stability, and motion in reduced dimensions. By offering fundamental insights into soliton-boundary energy landscapes, the topological magnetic soliton spring oscillator model lays the groundwork for deeper exploration of soliton behavior in confined systems, advancing the understanding of their dynamics in condensed matter physics and spintronic applications.

## Method

Micromagnetic simulations are conducted by mumax3^54^. The simulation parameters used for the hopfion are listed in Table 1. The values are corresponding to MnSi, include a saturation magnetization *M*_s_ = 1.51 × 10^5^ A/m, exchange constant *A* = 0.16 pJ/m, bulk Dzyaloshinskii-Moriya interaction (DMI) constant *D* = 0.117 mJ/m^2^, spin polarization of current density *p* = 0.6, perpendicular anisotropy constant *K* = 4.1 × 10^4^ J/m^3^, and nonadiabatic coefficient *β* = 0.2. Surface pinning was implemented using strong perpendicular magnetic anisotropy *K*_s_ = 0.5 mJ/m^23^.

**Table 1:** Simulation parameters used for hopfion.

Parameter	Value	Parameter	Value
*M*_S_	1.51 × 10^5^ A/m	*K*	4.1 × 10^4^ J/m^3^
*A*	0.16 pJ/m	*K*_s_	0.5 mJ/m^2^
*D*	0.117 mJ/m^2^	*β*	0.2
*p*	0.6	——	—

For skyrmion simulations, to avoid Hall effect, we adopt a cuboid box with dimensions 128 × 64 × 1 nm^3^ representing two antiferromagnetically coupled sublayers. The parameters used in the simulations are listed in Table 2, including a saturation magnetization *M*_s_ = 5.8 × 10^5^ A/m, exchange constant *A* = 15 pJ/m, surface Dzyaloshinskii-Moriya interaction (DMI) constant *D* = 3 mJ/m^2^, current density *j* = 1 × 10^11^ A/m^2^, spin polarization of current density *p* = 0.6, perpendicular anisotropy constant *K* = 8 × 10^5^ J/m^3^, and nonadiabatic coefficient *β* = 0.2^57,58^. The same parameters were applied to the domain wall simulations in a cuboid box with dimensions 96 × 48 × 1 nm^3^ except for the current density, which was set to *j* = 1 × 10^12^ A/m^2^.

**Table 2:** Simulation parameters used for skyrmion and domain wall.

Parameter	Value	Parameter	Value
*M*_S_	5.8 × 10^5^ A/m	*K*	8 × 10^5^ J/m^3^
*A*	15 pJ/m	*p*	0.6
*D*	3 mJ/m^2^	*β*	0.2

## Electronic supplementary material

Below is the link to the electronic supplementary material.

Supplementary Material 1

Supplementary Material 2

Supplementary Material 3

Supplementary Material 4

Supplementary Material 5

Supplementary Material 6

Supplementary Material 7

Supplementary Material 8

Supplementary Material 9

Supplementary Material 10

Supplementary Information

## Data Availability

The data that support the findings of this study are available from the corresponding author upon reasonable request.
