# Supplementary material for: A unified framework to explore soliton boundary interaction using topological magnetic soliton spring oscillators
Source: Sci Rep. 2025 Sep 30;15:33839. doi: 10.1038/s41598-025-05241-4 (PMC12484611; doi:10.1038/s41598-025-05241-4)
Supplement: Supplementary file 11 — Supplementary Information [file 41598_2025_5241_MOESM11_ESM.docx]

A Unified Framework to Explore Soliton Boundary Interaction Using Topological Magnetic Soliton Spring Oscillators

Shizhu Qiao^1,*^, Yan Zhou^2,‡^, Shishen Yan^3,†^,

Zhiyong Quan^4^, and Wenjia Yang^1^

^1^Department of Physics and Electronic Engineering, Jinzhong University, Jinzhong, 030619, China

^2^School of Science and Engineering, The Chinese University of Hong Kong, Shenzhen 518172, China

^3^School of Physics, State Key Laboratory of Crystal Materials, Shandong University, Jinan 250100, China

^4^Key Laboratory of Magnetic Molecules and Magnetic Information Materials of the Ministry of Education, Research Institute of Materials Science, Shanxi Normal University, Taiyuan, 030000, China

^*^Corresponding Author: [ryjqyears@gmail.com](mailto:ryjqyears@gmail.com)

^‡^Corresponding Author: [zhouyan@cuhk.edu.cn](mailto:zhouyan@cuhk.edu.cn)

^†^Corresponding Author: [shishenyan@sdu.edu.cn](mailto:shishenyan@sdu.edu.cn)

**SUPPLEMENTARY INFORMATION**

**Supplementary Code 1:** Mumax3 code for hopfion spring oscillator

**Supplementary Code 2:** Mumax3 code for skyrmion spring oscillator

**Supplementary Code 3:** Mumax3 code for domain wall spring oscillator

**Supplementary Movie 1:** Hopfion spring oscillator of size 128 × 64 × 16 nm^3^

**Supplementary Movie 2:** Skyrmion spring oscillator of size 128 × 64 ×1 nm^3^

**Supplementary Movie 3:** Hopfion explosion due to high current density and boundary repulsion.

**Supplementary Movie 4:** Skyrmion annihilation due to high current density

**Supplementary Movie 5:** Breathing of hopfion due to resonant excitation

**Supplementary Movie 6:** Hopfion spring oscillator of size 128 × 61 × 16 nm^3^

**Supplementary Movie 7:** Domain wall spring oscillator of size 96 × 48 × 1 nm^3^

**Equation for hopfion dynamics**

Starting from the Landau-Lifshitz-Gilbert (LLG) equation with the inclusion of the spin-transfer torque term,

$\frac{\text{d}m}{\text{d}t}=-\gamma\left[m\times B_{\text{eff}}\right]+\alpha\left[m\times\frac{\text{d}m}{\text{d}t}\right]+\frac{pa^{3}}{2e}\left[(j\cdot\nabla)m\right]-\frac{pa^{3}\beta}{2e}\left[m\times(j\cdot\nabla)m\right]$ (1)

Applied [***m*** ×] to both side of Equation 1, and using the vector identity$A\times\left[B\times C\right]=B\left(A\cdot C\right)-C\left(A\cdot B\right)$, one obtains:

$m\times\frac{\text{d}m}{\text{d}t}=\gamma B_{\text{eff}}-\alpha\frac{\text{d}m}{\text{d}t}+\frac{pa^{3}}{2e}m\times\left[(j\cdot\nabla)m\right]+\frac{pa^{3}\beta}{2e}(j\cdot\nabla)m$ (2)

To express the time derivative of the magnetization in terms of collective coordinates $\xi\left(t\right)=\left\{\xi_{0},\xi_{1},...\right\}$, we define ^56^. Applying $\left(\partial m/\partial\xi_{i}\cdot\right)$ to both side of Equation 2, we obtain:

$\frac{\partial m}{\partial\xi_{i}}\cdot\left[m\times\frac{\partial m}{\partial\xi_{j}}\mathop{\xi_{j}}\limits^{.} \right]=\frac{\partial m}{\partial\xi_{i}}\cdot\gamma B_{\text{eff}}-\alpha\frac{\partial m}{\partial\xi_{i}}\cdot\frac{\partial m}{\partial\xi_{j}}\mathop{\xi_{j}}\limits^{.} +\frac{pa^{3}}{2e}\frac{\partial m}{\partial\xi_{i}}\cdot m\times\left[(j\cdot\nabla)m\right]+\frac{\partial m}{\partial\xi_{i}}\cdot\frac{pa^{3}\beta}{2e}(j\cdot\nabla)m$ (3)

where Einstein summation convention is used. Using the identity $A\cdot\left[B\times C\right]=B\cdot\left[C\times A\right]$ and the definition of the effective field $B_{\text{eff}}=-\left(1/M_{\text{s}}\right)\delta U/\delta m$, we get:

$m\cdot\left[\frac{\partial m}{\partial\xi_{j}}\times\frac{\partial m}{\partial\xi_{i}}\right]\mathop{\xi_{j}}\limits^{.} =-\gamma\frac{1}{M_{\text{s}}}\frac{\partial m}{\partial\xi_{i}}\cdot\frac{\delta U}{\delta m}-\alpha\frac{\partial m}{\partial\xi_{i}}\cdot\frac{\partial m}{\partial\xi_{j}}\mathop{\xi_{j}}\limits^{.} +\frac{pa^{3}}{2e}\frac{\partial m}{\partial\xi_{i}}\cdot m\times\left[(j\cdot\nabla)m\right]+\frac{pa^{3}\beta}{2e}\frac{\partial m}{\partial\xi_{i}}\cdot(j\cdot\nabla)m$(4)

Integrating over the volume of the magnet, and taking $\int\delta U/\delta m\cdot\partial m/\partial\xi_{i}\text{d}V=\partial U/\partial\xi_{i}$ into consideration, yields

$\int m\cdot\left[\frac{\partial m}{\partial\xi_{j}}\times\frac{\partial m}{\partial\xi_{i}}\right]\mathop{\xi_{j}}\limits^{.} \text{d}V=-\gamma\frac{1}{M_{\text{s}}}\frac{\partial U}{\partial\xi_{i}}-\alpha\int\frac{\partial m}{\partial\xi_{i}}\cdot\frac{\partial m}{\partial\xi_{j}}\mathop{\xi_{j}}\limits^{.} \text{d}V+\frac{pa^{3}}{2e}\int\frac{\partial m}{\partial\xi_{i}}\cdot m\times\left[(j\cdot\nabla)m\right]\text{d}V+\frac{pa^{3}\beta}{2e}\int\frac{\partial m}{\partial\xi_{i}}\cdot(j\cdot\nabla)m\text{d}V$ (5)

Assuming the entire hopfion moves with the same velocity, the collective coordinate $\mathop{\xi_{j}}\limits^{.}$ can be factored out of the integration. Since the deformation of the hopfion is weak (less than 10%), it is reasonable to ignore collective coordinates related to the deformation^56^. Additionally, considering the hopfion moves mainly along the *x*-direction, we obtain:

$\frac{pa^{3}\beta j}{2e}\int\frac{\partial m}{\partial x}\cdot\frac{\partial m}{\partial x}\text{d}V-\alpha\int\frac{\partial m}{\partial x}\cdot\frac{\partial m}{\partial x}\text{d}V\mathop{x}\limits^{.} -\gamma\frac{1}{M_{\text{s}}}\frac{\partial U}{\partial x}=0$ (6)

Noting that $\Gamma_{xx}=\int\frac{\partial m}{\partial x}\cdot\frac{\partial m}{\partial x}\text{d}V$, Equation (2) in the main text can be derived.

**Breathing mode of hopfion**


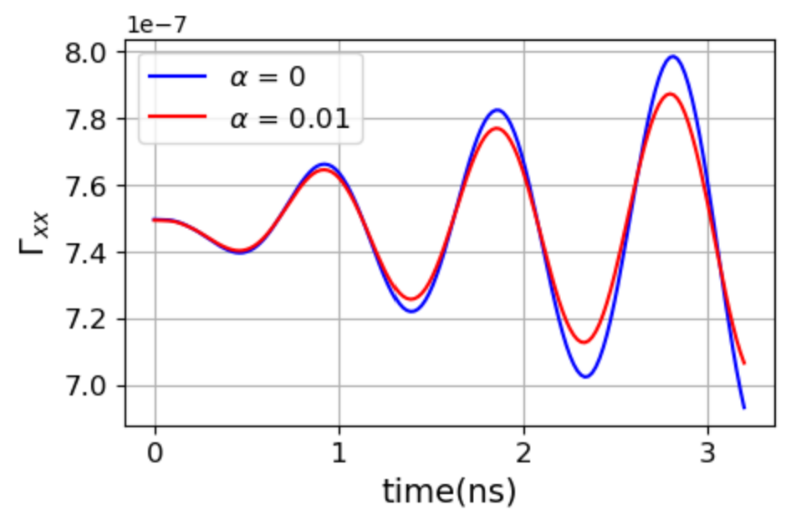


Supplementary Figure S1 Breathing mode-induced Γ*_xx_* variation of hopfion under a microwave field ***B*** = 5 × 10^-4^ sin(2π*ft*) ***e****_z_* with *f* = 1.06 GHz of two cases, *α* = 0 and *α* = 0.01

The breathing mode induced by the microwave field ***B*** = 5 × 10^-4^ sin(2π*ft*)***e****_z_* is illustrated in Supplementary Fig. S1, where the time evolution of Γ*_xx_* for the two cases, *α* = 0 and *α* = 0.01, are depicted. It is evident that the amplitude in Supplementary Fig. S1 is much larger than that shown in Fig. 3(a) in the main text, due to resonance excitation. Additionally, the amplitude in the case of *α* = 0.01 is noticeable smaller than that in case of *α* = 0, supporting the conclusion made about Fig. 4(a) in the main text. Please refer to Supplementary Movie 5 for more details.

**Results of hopfion spring oscillator of dimensions 256 × 64 × 16 nm³**


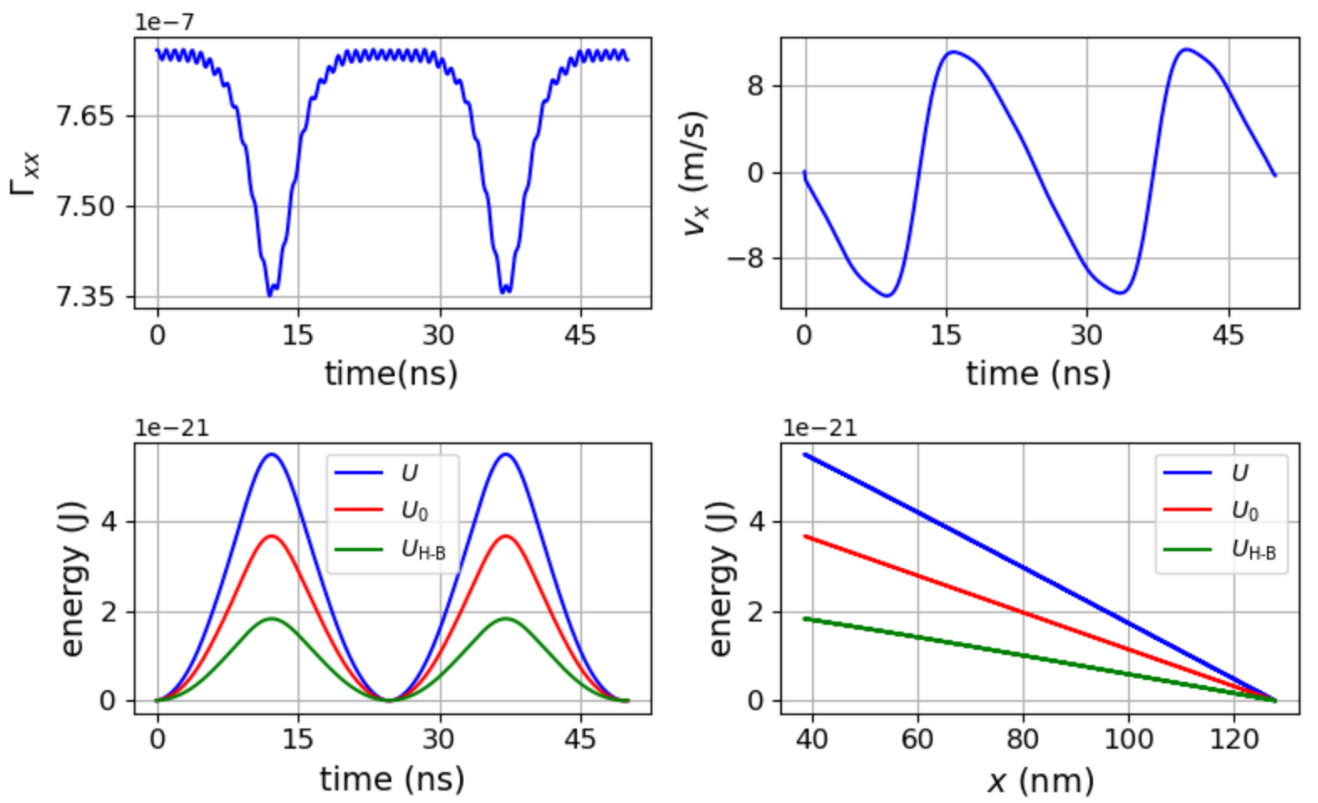


(b)

(a)

(c)

(d)

Supplementary Figure S2 Numerical results of hopfion spring oscillator of dimensions 256 × 64 × 16 nm^3^: Dependence of (a) Γ*_xx_* and velocity of hopfion center (b) on time. Dependence of *U*, *U*_0_, and *U*_H-B_, on time (c), and *x*-coordinate of hopfion center (d).

Simulation results of hopfion spring oscillator of dimensions 256 × 64 × 16 nm^3^ are depicted in Supplementary Fig. S2. In this simulation, current density *j* = 2 × 10^9^ A/m^2^ is adopted, to avoid the destruction of hopfion. The time dependence of Γ*_xx_*, shown in Supplementary Fig. S2 (a), exhibits a variation about 5%, similar to that of dimensions 128 × 64 × 16 nm^3^. The velocity of hopfion center shown in Supplementary Fig. S2 (b) and time dependence of energy in Supplementary Fig. S2 (c) indicate that the oscillation in this circumstance deprives from harmonic oscillation. Although this deprivation, a linear potential still occurs in this circumstance, as seen in Supplementary Fig. S2 (d).

**Results of hopfion spring oscillator with *α* = 0.05**


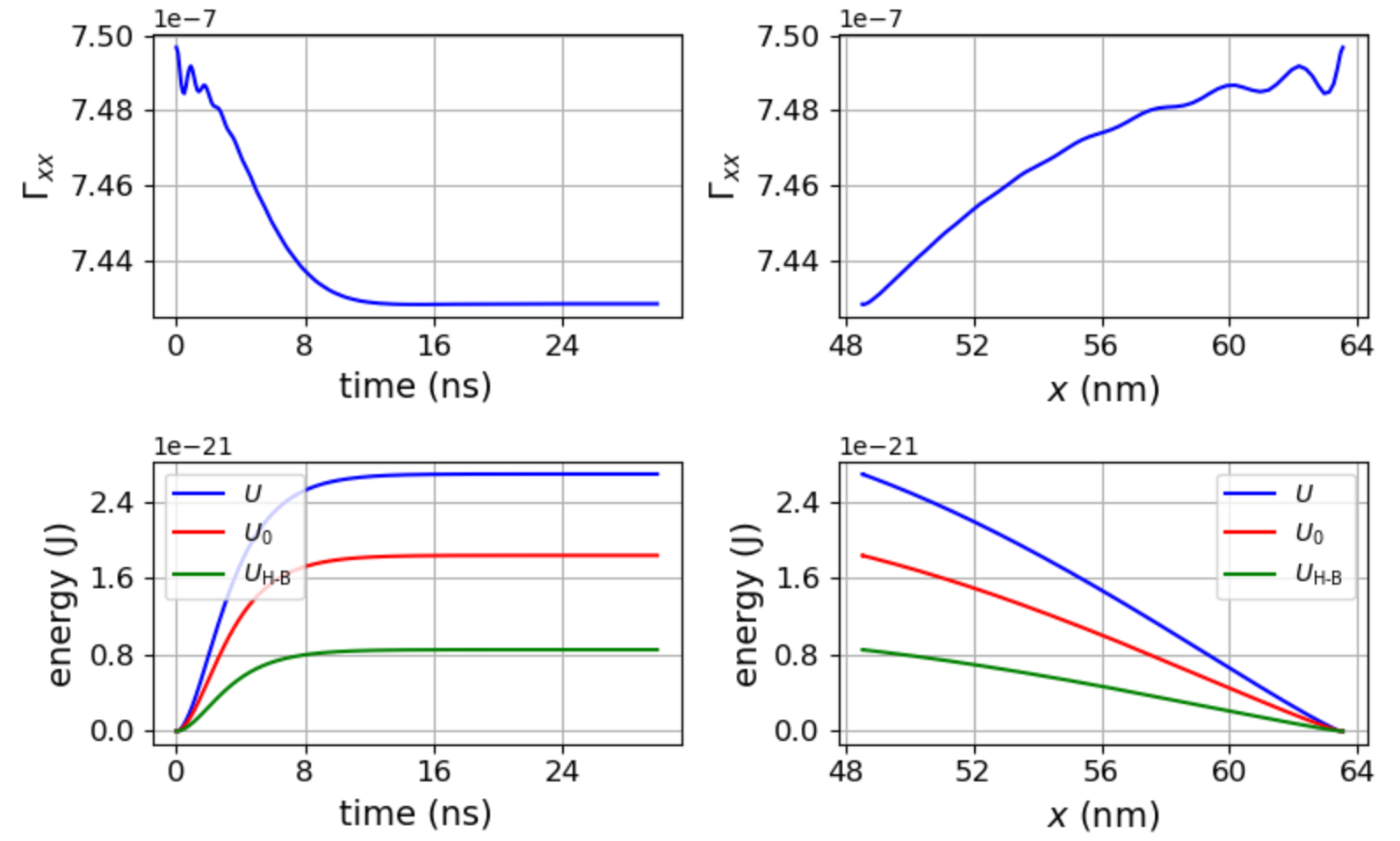


(b)

(a)

(c)

(d)

Supplementary Figure S3 Numerical results of hopfion spring oscillator in circumstance of *α* = 0.05: Dependence of Γ*_xx_* on time (a), and *x*-coordinate of hopfion center (b). Dependence of *U*, *U*_0_, and *U*_H-B_, on time (c), and *x*-coordinate of hopfion center (d). Linear dash lines are plotted in (d) for visual assistance.

Simulation results for the case with *α* = 0.05 is depicted in Supplementary Fig. S3. With this large damping factor, both Γ*_xx_* and energy quickly reach stable values, as shown in Supplementary Fig. S3 (a) and S3 (c). In this scenario, the variation of Γ*_xx_* is significantly smaller—around 1%—as indicated in Supplementary Fig. S3 (a) and S3 (b), compared to 4% variation observed in the *α* = 0 circumstance. Furthermore, the linear dash lines in Supplementary Fig. S3 (d) show that *U*_H-B_ ​no longer exhibits linear behavior in this high-damping scenario.

**Results of hopfion in cuboid box without boundaries perpendicular to *x*-axis and the method for calculating *U*₀**


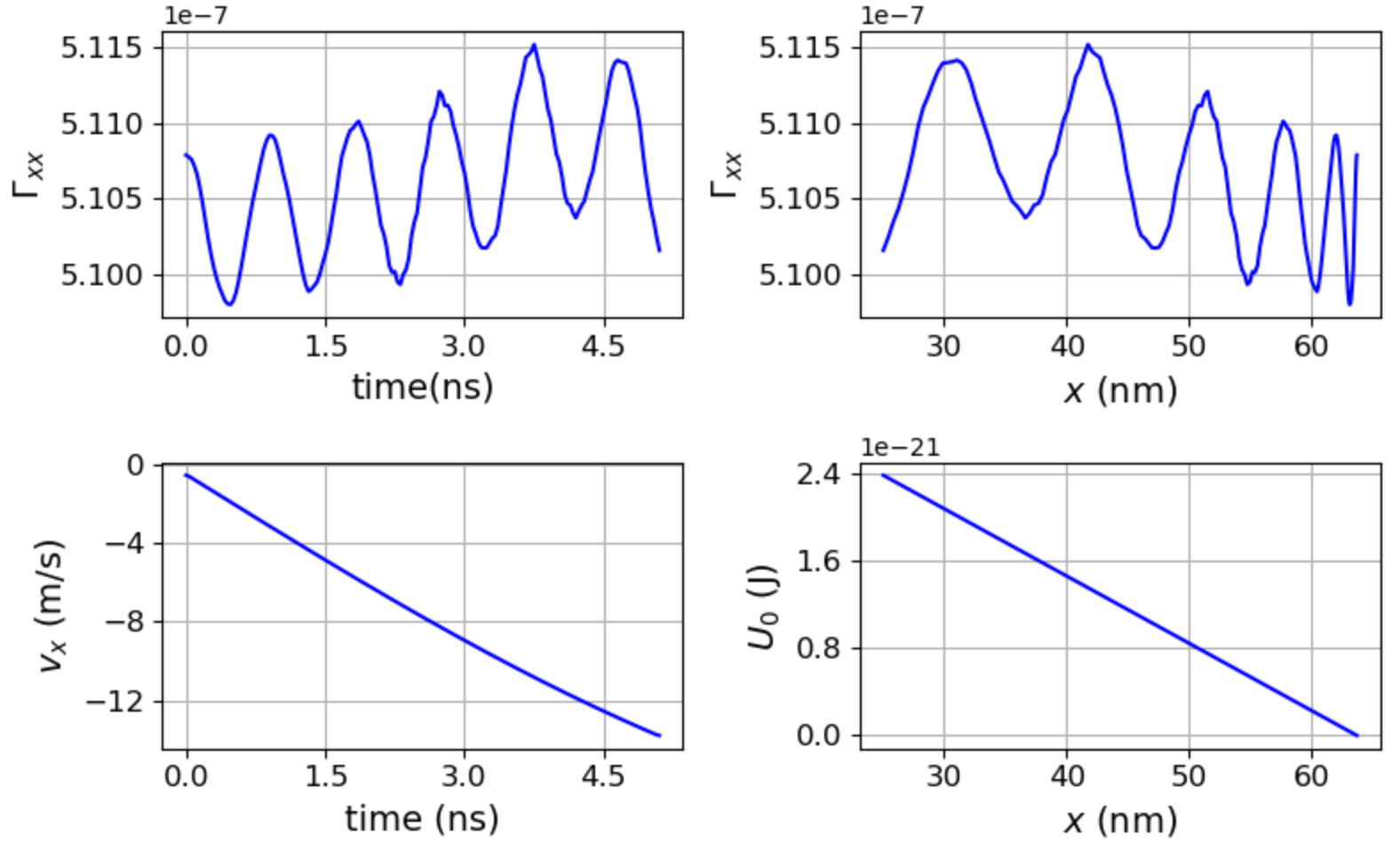


(b)

(a)

(c)

(d)

Supplementary Figure S4 Numerical results of a hopfion in cuboid box without boundaries perpendicular to *x*-axis: time (a) and *x*-coordinates (b) dependence of Γ*_xx_*, (c) time dependence of velocity of hopfion center, (d) *x*-coordinates dependence of *U*_0_.

Using periodic boundary conditions along the *x*-axis, the simulation results of a hopfion in a cuboid box without boundaries perpendicular to the *x*-axis are displayed in Supplementary Fig. S4. In this scenario, the variation of Γ*_xx_* is primarily caused by the breathing mode, as illustrated in Supplementary Fig. S4 (a) and S4 (b), which exhibit a frequency of approximately 1.06 GHz. The velocity of the hopfion, shown in Supplementary Fig. S4 (c), displays a monotonically increasing trend due to the influence of spin-transfer torque without boundary repulsion along the *x*-axis. Since there are no boundaries perpendicular to the *x*-axis, there is no hopfion-boundary interaction energy *U*_H-B_, and the total energy *U* is simply *U*_0_ in this case. The dependence of *U*_0_ on the *x*-coordinate of the hopfion center is depicted in Supplementary Fig. S4 (d), where *U*_0_ at *x* = 64 nm is set to zero.

To calculate *U*_0_ in circumstances with boundaries perpendicular to *x*-axis, we first apply periodic boundary conditions along the *x*-axis to determine Γ*_xx_* without the impact of boundaries perpendicular to the *x*-axis, denoted as $\Gamma_{xx}^{0}$. As a first-order approximation, *U*_0_ can be calculated using the following equation:

$U_{0}\left(x\right)=\int_{x_{0}}^{x} \left(\frac{pa^{3}\beta j}{2e}-\alpha v_{x}\right)\frac{\Gamma_{xx}^{0}M_{\text{s}}}{\gamma}\text{d}x$, (7)

where *v_x_* is the velocity in the presence of boundaries perpendicular to the *x*-axis.

**Frequency and relaxation of the hopfion spring oscillator when *α* ≠ 0**


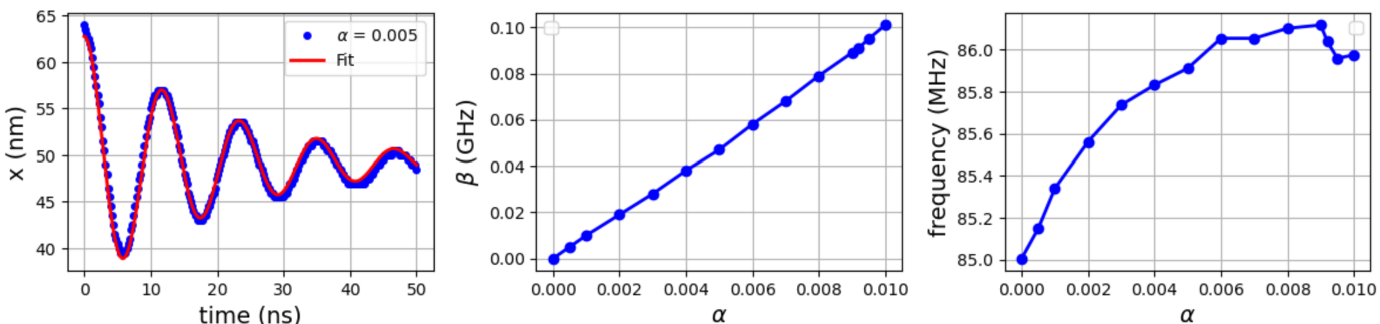


(a)

(b)

(c)

Supplementary Figure S5: (a) Evolution of *x*-coordinate of hopfion center when α = 0.005 and *j* = 4×10^9^ A/m^2^, blue points are data extracted from simulation, and the red curve is fitted curve with damped cosine function . Dependence of (b) fitted *β* and (c) frequency on Gilbert damping factor.

As shown in Figure S5(a), the temporal evolution of the hopfion center is fitted using a damped cosine function . The fitting parameter *β* as a function of *α* is plotted in Figure S5(b), showing an approximately linear dependence. This result is consistent with the analytical prediction from Equation (6) and the damped harmonic oscillator model (Equation 7), where damping terms are proportional to *α* and *β*, respectively.

$\frac{\text{d}^{2}x}{\text{d}t^{2}}+2\beta\frac{\text{d}x}{\text{d}t}+\omega_{0}^{2}x=0$ (7)

Interestingly, the oscillation frequency as a function of *α* is presented in Figure S5(c). It shows that the frequency increases slightly with increasing α, which deviates from the classical expectation $\omega=\sqrt{\omega_{0}^{2}-\beta^{2}}$ for a damped harmonic oscillator. This deviation highlights the nonlinear nature of hopfion dynamics governed by Equation (6), in contrast to the linear model described by Equation (7).

The irregularity observed for *α* near 0.01 (marked by the red circle in Figure S5(c)) arises from the limited number of oscillation periods available for fitting. Specifically, when *α* approaches 0.01, only 2 to 3 oscillation periods remain before the motion is fully damped, as seen in Figure 2(b) in the main text, leading to larger fitting errors and frequency fluctuations.

**Enhancing one-dimensionality of the hopfion spring oscillator by reducing *l_y_***

As shown in Supplementary Movie 1, the soliton in the hopfion spring oscillator with dimensions 128 × 64 × 16 nm^3^ exhibits weak motion along the *y*-axis due to the Hall effect. This weak motion can be further suppressed by reducing *L_y_*, as demonstrated in Supplementary Movie 6, which depicts a hopfion spring oscillator with dimensions 128 × 61 × 16 nm^3^.
